# Multidimensional modulation of systemic immune by neurosurgical tumor resection in patients with brain tumors

**DOI:** 10.1002/iid3.703

**Published:** 2022-09-15

**Authors:** Jia‐Wei Wang, Hong‐Liang Wang, Qi Liu, Ke Hu, Liujiazi Shao, Jing‐Hai Wan

**Affiliations:** ^1^ Department of Neurosurgery, National Cancer Center/National Clinical Research Center for Cancer/Cancer Hospital Chinese Academy of Medical Sciences and Peking Union Medical College Beijing People's Republic of China; ^2^ Department of Neurosurgery The Second Affiliated Hospital of Anhui Medical University Hefei People's Republic of China; ^3^ Department of Anesthesiology, Beijing Friendship Hospital Capital Medical University Beijing People's Republic of China

**Keywords:** brain tumor, immune remodeling, neurosurgical tumor resection, systemic immune

## Abstract

**Objectives:**

Immune perturbation induced by tumor burden has been showed as the hallmark of brain tumors. To date, the vast majority of studies have focused heavily on local immune responses in the tumor microenvironment. Little is known about how the systemic immune macroenvironment is modulated by neurosurgical tumor resection in patients with brain tumors.

**Method:**

Medical records from patients with brain tumors admitted to the Department of Neurosurgery at the National Cancer Center, Cancer Hospital of Chinese Academy of Medical Sciences between January 2021 and March 2022 were retrospectively reviewed. Forty‐nine patients who have lymphocyte subsets, serum immunoglobulins, C‐reactive protein, and complements levels before neurosurgical tumor resection and at least once test after surgery were included into the final analysis.

**Results:**

Postoperative CD3+ lymphocytes, CD4+ lymphocytes and CD4+ /CD8+ lymphocyte ratio presented bi‐phasic changes, which indicated an initial decrease and a subsequent increase after neurosurgical tumor resection. Moreover, neurosurgical tumor resection induced a decrease in natural killer lymphocytes and an increase in B lymphocytes that persisted through the entire observation period after surgery. Meanwhile, significant changes in humoral immunity characterized by a decrease in immunoglobulins (IgA, IgG, and IgM) levels and an increase in the CRP level occurred after neurosurgical tumor resection. In addition, patients with postoperative infection complication had a lower preoperative CD4+ /CD8+ lymphocyte ratio.

**Conclusions:**

These findings provide evidence that either cellular immunity or humoral immunity can be remodeled by neurosurgical tumor resection, and patients with disturbed systemic immunity have increased risk of infection after surgery.

## Graphical abstract

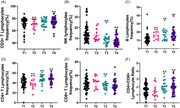

## 1 INTRODUCTION

During the past decades, the extensively bidirectional interaction between the central nervous system (CNS) and the immune system has been highlighted in the clinical and preclinical scenario.^1^ The CNS‐mediated immunosuppression may occur following a variety of acute and chronic CNS insults such as traumatic brain injury,^2^ stroke,^3^ and brain tumors.^4^, ^5^, ^6^ It has been realized that these CNS insults can induce a disturbance of the normally well‐balanced interplay between the CNS and immune system, and eventually lead to brain‐specific secondary immunodeficiency that is nominated as CNS injury‐induced immunodepression syndrome by Christian Meisel and his co‐authors.^1^ Moreover, based on three distinct models of brain cancers including GL261 glioma, B16 melanoma, and a spontaneous model of diffuse intrinsic pontine glioma, Ayasoufi and his colleagues recently have also demonstrated that the CIDS is not unique to specific brain tumor itself, but rather occurs in response to brain injury.^7^ These pieces of knowledge should be integrated into the treatment strategies in the context of CNS diseases to improve the outcome.^8^

Neurosurgical tumor resection, as well as chemotherapy and radiation, are the mainstays of the therapeutic strategies in brain tumors, all of which may result in perturbations in immunity.^9^, ^10^ Thus, understanding how the host respond to these different cancer therapies is important for designing strategies that augment rather than impede antitumor immune responses, including optimal timing, dosing, or combinations, especially when it comes to the era of immunotherapy that has revolutionized cancer therapy.^11^ To the best of our knowledge, to date, there are few studies that have fully characterized the relationship between neurosurgical tumor resection and systemic immune landscape including cellular immune and humoral immune in patients with brain tumors.

Actually, our understandings about the effects of surgery on the systemic immune are mainly from peripheral cancers instead of brain tumors, which have shown that surgery may result in both detrimental and beneficial effects on the systemic immune system.^9^, ^11^, ^12^ For example, Allen et al. have found that surgical tumor resection in several mice models of peripheral cancer can reverse the systemic immune changes that occurs across mice cancer models and time, and the alterations in immunosuppression are characterized by dampened responses to orthogonal challenges including reduced T cell activation during viral or bacterial infection.^13^ Another study showed surgery suppressed natural killer (NK) cell function and prevented them from removing experimental lung metastases and the enhanced lung metastases in tumor‐bearing mice was observed when surgically stressed NK cells are transferred into NK‐deficient mice.^14^ Thus, it is particularly important to consider the dual effects of surgery, depict the whole picture of immunosuppression in patients with brain tumors undergoing surgery, and further explore potential interventions to restore immune function.

The present study aimed to investigate the time course and alterations in systemic immunity modulated by neurosurgical tumor resection in the patients with brain tumors based on the analysis of the cellular immune and humoral immune biomarkers across time. Furthermore, we tested the hypothesis that infection after neurosurgical tumor resection was associated with the perturbations in the systemic immune before surgery.

## 2 MATERIALS AND METHODS

### 2.1 Study population

**Figure 1:**
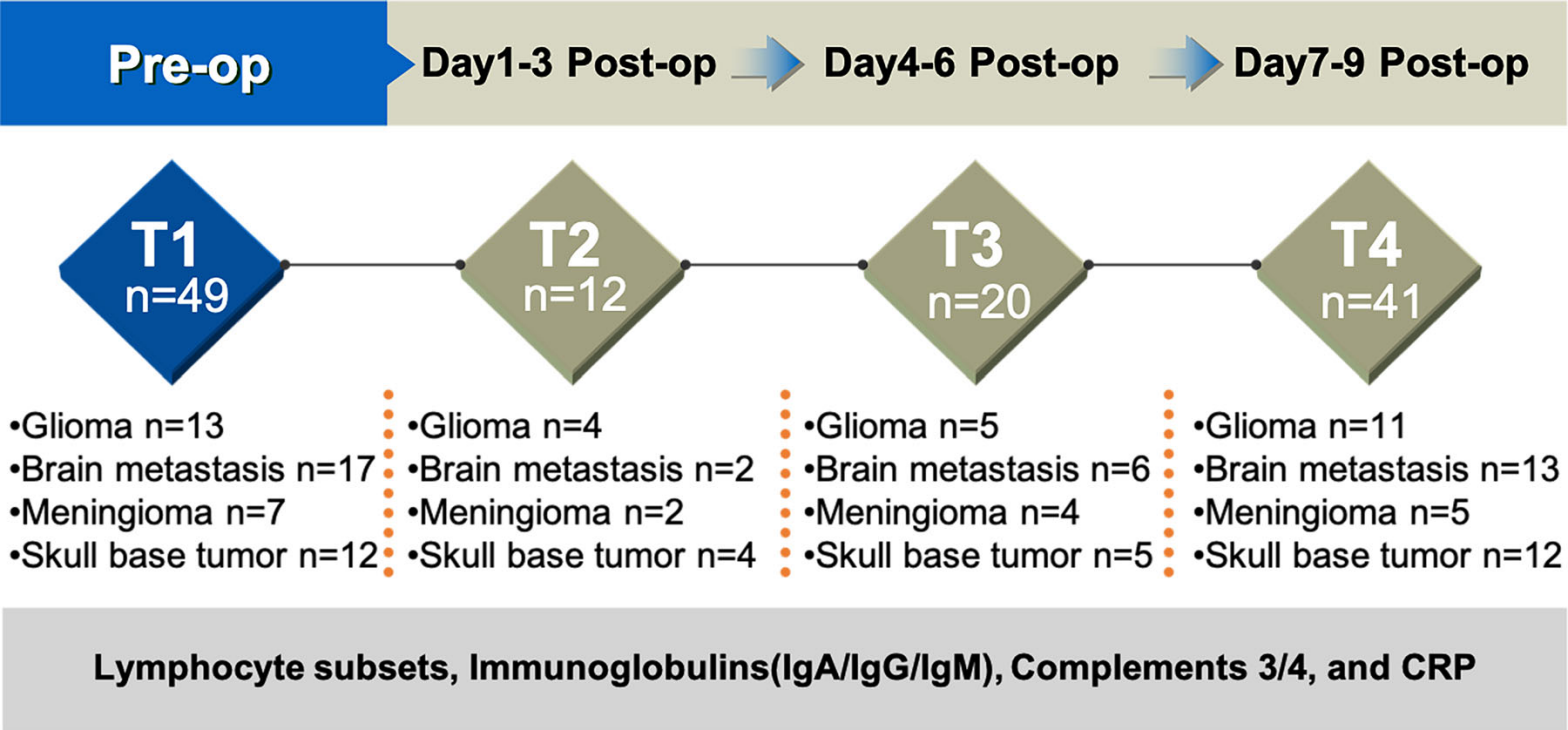
Flow chart of the study and demographic data of included patients. Forty‐nine patients who have lymphocyte subsets, serum immunoglobulins, C‐reactive protein, and complements levels before neurosurgical tumor resection and at least one test after surgery were included into the final analysis. CRP, C‐reactive protein; Post‐op, postoperative; Pre‐op, preoperative.

This was a retrospective study. This research was approved by the Ethics Committee of the Cancer Hospital, Chinese Academy of Medical Sciences (No.22/052‐3253) and was performed in accordance with the World Medical Association Declaration of Helsinki. Informed content was obtained from all the participants. Medical records from patients with brain tumors admitted to the Department of Neurosurgery at the National Cancer Center (NCC), Cancer Hospital of Chinese Academy of Medical Sciences between January 2021 and March 2022 were retrospectively reviewed. Patients who have cellular immune and humoral immune tests before neurosurgical tumor resection and at least one test after surgery were included into the final analysis (Figure 1). Other data concerning the demographic parameters of each patient were also retracted from the medical records, including age and gender of patients, Karnofsky Performance Status (KPS) scores, pathology type, and treatment information.

### 2.2 Treatment strategies

All the patients in the present were treated by the same team led by senior authors (Dr. Jing‐Hai Wan and Dr. Jia‐Wei Wang) and underwent elective neurosurgery (craniotomy for brain tumors). All the patients received the same perioperative antibiotic prophylaxis (Cefuroxime). Patients were defined as having an infectious complication if a systemic response to infection was clinically evident (two or more of the following signs: peripheral white blood cell count of less than 4 × 10^9^ cells/L or more than 10 × 10^9^ cells/L; fever; elevated and increasing C‐reactive protein (CRP) or procalcitonin concentration; meningeal irritation symptoms; cerebrospinal fluid (CSF) analysis showing significantly increased white blood cells (especially multinucleated cells), and decreased sugar content; and positive culture of pathogenic microorganisms from sputum, blood, urine, or CSF, along with clinical and imaging evidence of an infection site such as enhanced cranial magnetic resonance imaging.

### 2.3 Flow cytometry for lymphocyte subsets

To evaluate the effects of neurosurgical tumor resection on cellular immunity, lymphocyte subsets were examined by flow cytometric analysis using commercial kits as described previously by our team.^2^ A single platform with a lyse‐no‐wash procedure by the BD FACS Canto II flow cytometer (BD Biosciences) was used with two sets of four‐color monoclonal antibody combinations (BD Biosciences), which included BD Multitest CD3 (FITC)/CD8 (PE)/CD45 (PerCP)/CD4 (APC), and BD Multitest CD3 (FITC)/CD16+ CD56 (PE)/CD45 (PerCP)/CD19 (APC). In brief, these two sets of four‐color monoclonal antibody combinations were labeled as tubes 1 and 2, respectively. Then 50  μl of blood samples was incubated with 20 μl of the monoclonal antibodies. Red cells were lysed with the BD multitest IMK kit (BD Biosciences). Finally, the preparation was analyzed on BD FACS Canto II flow cytometer with the BD Diva software packages (BD Biosciences). Lymphocytes were identified by their strong CD45 expression and low side scatter. T cells, T helper cells, cytotoxic T cells, NK cells and B lymphocytes were identified by expression of CD3+ , CD3+CD4+, CD3+CD8+, CD3‐CD16&56+, and CD3‐CD19+, respectively.

### 2.4 Rate immune scatter turbidimetry for humoral immunity

To evaluate the effects of neurosurgical tumor resection on humoral immunity, serum immunoglobulins (IgA, IgG, and IgM), CRP, and complements (C3 and C4) levels were determined by rate immune scatter turbidimetry using commercial kits on the Immage 800® Immunochemistry System (Beckman Coulter).^2^ Reference ranges for healthy adults, as established in our hospital, were between 1.0 and 4.2 g/L for IgA, between 8.6 and 17.4 g/L for IgG, between 0.5 and 2.8 g/L for IgM, between 0.0 and 0.6 mg/dl for CRP, between 79 and 152 mg/dl for C3, and between 16.0 and 38.0 mg/dl for C4.

### 2.5 Statistical analysis

Prism version 9.0 (GraphPad Software) was used for the statistical analysis and graphing. The data were presented as number or mean ± SD. Lymphocyte subsets, serum immunoglobulins (IgG, IgA, and IgM), CRP, and complements (C3 and C4) levels before surgery and different time points after surgery were analyzed using a paired *t*‐test. And unpaired *t*‐test was used to compare the preoperative lymphocyte subsets and humoral immunity between the infection and non‐infection groups. Statistical significance was accepted with *p* < .05.

## 3 RESULTS

As shown in Figure 1, 49 patients with brain tumors (age: 50.98 ± 13.11 years) were enrolled into the study after meeting the criteria, with 26 males and 23 females. In the entire group, according to the 2016 WHO Classification of Tumors of the Central Nervous system (fourth revised version), glioma constitutes 26.53% (WHO Grade IV: *n* = 7, Grade III: *n* = 1, Grade II: *n* = 5), brain metastasis 34.69% (*n* = 11 from lung cancer, *n* = 3 from colorectal cancer, *n* = 1 from breast/kindey/esophageal carcinoma), convexity and falcine meningioma (WHO Grade I: *n* = 6, Grade II and III: *n* = 1) 14.29%, and skull base tumors 24.49% (meningioma: *n* = 5 [WHO Grade I: *n* = 3, Grade II: *n* = 2], sarcoma: *n* = 3, schwannoma: *n* = 3, pleomorphic adenoma: *n* = 1). Lymphocyte subsets, serum immunoglobulins (IgG, IgA, and IgM), CRP, and complements (C3 and C4) levels were tested in all the included patients before surgery, and the time‐points of these tests after surgery could be divided into three phases: Days 1–3, 4–6, and 7–9 postoperatively (Figure 1).

### 3.1 Effects of neurosurgical tumor resection on the cellular immunity

**Figure 2:**
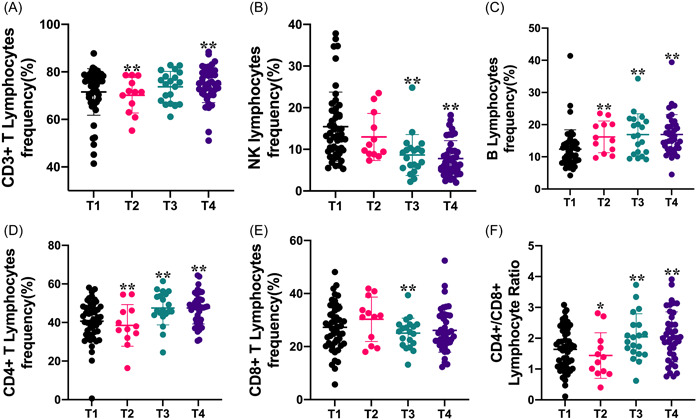
Effects of neurosurgical tumor resection on the cellular immunity assessed by peripheral blood lymphocyte subsets. Postoperative CD3+ lymphocytes (A), CD4+ lymphocytes (D), and CD4+/CD8+ lymphocyte ratio (F) presented bi‐phasic changes, which indicated an initial decrease on Days 1–3 after surgery and a subsequent increase on Days 4–6 and 7–9 after surgery. Moreover, neurosurgical tumor resection induced a decrease in NK lymphocytes (B) and an increase in B lymphocytes (C) that persisted through the entire observation period after surgery. T1, preoperatively; T2, Days 1–3; T3, Days 4–6, and T4, Days 7–9 postoperatively. *p < .05, **p < .01 compared with preoperative levels.

In the present study, lymphocyte subsets were determined by flow cytometry to evaluate the effects of neurosurgical tumor resection on cellular immunity (Figure 2). As shown in Figure 2A, CD3+ T lymphocytes frequency decreased significantly at Days 1–3 after surgery in comparison with the one before surgery, and then recovered gradually. At Days 7–9 after surgery, CD3+ T lymphocytes frequency had become significantly higher than the one before surgery. As for the NK lymphocytes, the decrease in NK lymphocyte frequency persisted during the entire observation period after surgery, and became significant at Days 4–6 and 7–9 following surgery compared with the one before surgery (Figure 2B). In contrast, the significant increase in the B lymphocytes frequency was found thorough the entire observation period after surgery in comparison with the one before surgery (Figure 2C). Furthermore, data in Figure 2D,F indicated both CD4+ T lymphocytes frequency and CD4+/CD8+ lymphocyte ratio after surgery had similar bidirectional changes, which showed initially significant decrease early after surgery and then significantly increase at Days 4–6 and 7–9 after surgery. The decrease in CD8+ T lymphocytes frequency was significantly only at Days 4–6 after surgery in comparison with the one before surgery (Figure 2E).

### 3.2 Effects of neurosurgical tumor resection on the humoral immunity

**Figure 3:**
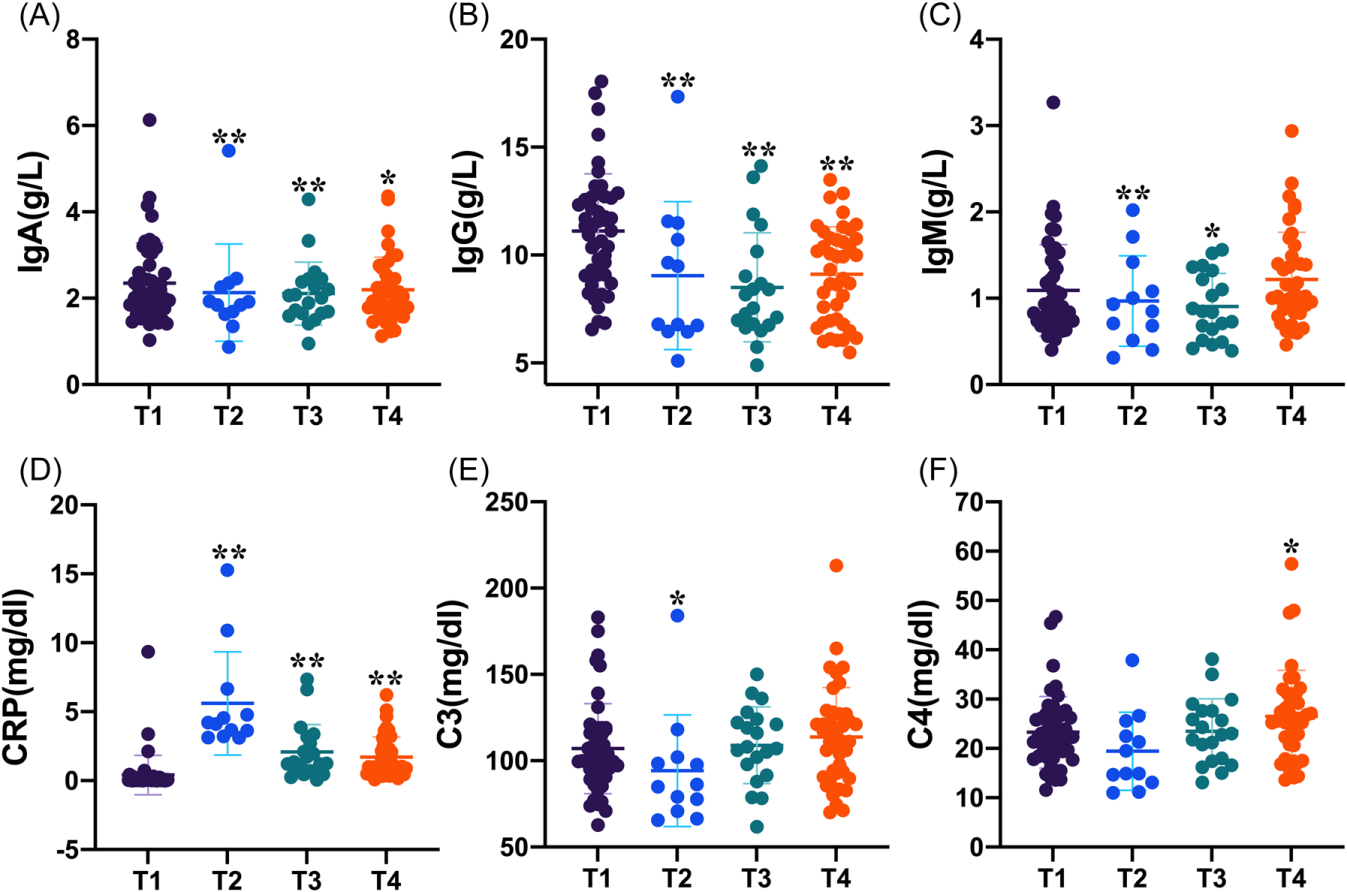
Effects of neurosurgical tumor resection on the humoral immunity assessed by serum immunoglobulins, CRP, and complements levels. significant changes in humoral immunity characterized by a decrease in immunoglobulins (IgA, IgG, and IgM) levels (A–C) and an increase in the CRP level (D) occurred after neurosurgical tumor resection. T1, preoperatively; T2, Days 1–3, T3, Days 4–6, and T4, Days 7–9 postoperatively. CRP, C‐reactive protein. *p < .05, **p < .01 compared with preoperative levels.

In the present study, serum immunoglobulins (IgA, IgG, and IgM), CRP, and complements (C3 and C4) levels were determined to evaluate the effects of neurosurgical tumor resection on humoral immunity (Figure 3). As shown in Figure 3A–C, patients with neurosurgical tumor resection showed significant decrease in both IgA and IgG levels thorough the entire observation period after surgery and in IgM level on Days 1–3 and 4–6 after surgery in comparison with the one before surgery. In contrast, there were significant increase in the CRP levels after surgery, especially on Days 1–3 postoperatively (Figure 3D). As for the complements C3 and C4 (Figure 3E,F), complements C3 showed a significant decrease on Days 1–3 after surgery and then gradually recovered to the normal level, while the complements C4 increased significantly on Days 7–9 after surgery.

### 3.3 Systemic immunity and infectious complications after surgery

**Figure 4:**
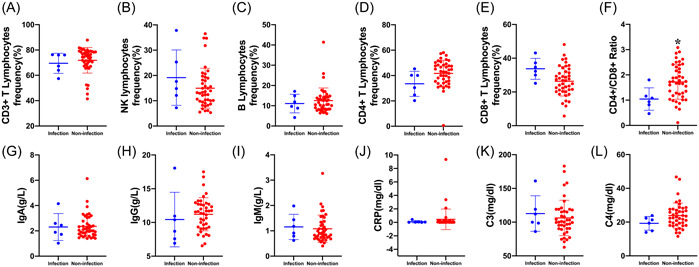
Systemic immunity and infectious complication after surgery. Patients with postoperative infection complication had a significantly lower preoperative CD4+/CD8+ lymphocyte ratio (F). CRP, C‐reactive protein. *p < .05 compared with preoperative levels.

To evaluate the systemic immune on the incidence of infectious complications after surgery, the preoperative lymphocyte subsets, serum immunoglobulins (IgG, IgA, and IgM), CRP, and complements (C3 and C4) levels in patients with postoperative infection and without postoperative infection were compared (Figure 4). A total of 6 (12.24%) among 49 patients had a documented infection during the postoperative period, and all were treated successfully with empiric antibiotic treatment. As shown in Figure 4, the preoperative CD4+/CD8+ lymphocyte ratio in patients with infection significantly decreased in comparison with the one without infection (1.05 ± 0.44 vs. 1.72 ± 0.69). And there were no significant differences with regard to lymphocyte frequency (CD3+ , CD4+ , CD8+ , NK, and B lymphocytes), serum immunoglobulins (IgG, IgA, and IgM), CRP, and complements (C3 and C4) levels before surgery in the patients with or without infection.

## 4 DISCUSSION

This is the first study to follow serial lymphocyte subsets, serum immunoglobulins (IgG, IgA and IgM), CRP, and complements (C3 and C4) levels in patients with brain tumors treated with neurosurgical tumor resection and to correlate the findings with postoperative infection complication. The main findings are as follows: (1) Postoperative CD3+ lymphocytes, CD4+ lymphocytes, and CD4+/CD8+ lymphocyte ratio presented bi‐phasic changes, which indicated an initial decrease on Days 1–3 after surgery and a subsequent increase on Days 4–6 and 7–9 after surgery. (2) Neurosurgical tumor resection induced a decrease in NK lymphocytes and an increase in B lymphocytes that persisted through the entire observation period after surgery. (3) Significant changes in humoral immunity characterized by a decrease in immunoglobulins (IgA, IgG, and IgM) levels and an increase in the CRP level occurred after neurosurgical tumor resection. (4) Patients with postoperative infection complication had a lower preoperative CD4+ /CD8+ lymphocyte ratio. These findings provide evidence that either cellular immunity or humoral immunity can be remodeled by neurosurgical tumor resection, and patients with disturbed systemic immunity have an increased risk of infection after surgery.

Immune perturbation induced by tumor burden has been showed as the hallmark of tumors, especially malignant tumors, which is associated with tumorigenesis, tumor growth, cancer progression, and patient survival.^8^, ^9^, ^10^ In the specific patients with brain tumors in our study, immune suppression has been extensively explored in preclinical and clinical research,^4^, ^6^, ^7^, ^15^, ^16^ which involves several mechanisms including immunosuppressive factors/cytokines secreted by tumor cells, suppressive surface markers expressed on the tumor cells, M2 phenotypic transformation in tumor‐associated macrophages/microglia, expansion of regulatory T lymphocyte and myeloid‐derived suppressor cell, and tumor‐induced lymphocyte abnormality. However, to date, the vast majority of studies in the cancer area have focused heavily on local immune responses in the tumor microenvironment (TME).^17^, ^18^ Systemic immune landscape beyond TME remains to be fully determined,^19^, ^20^, ^21^ especially in the patients with brain tumors needing neurosurgical resection. Moreover, dynamic assessing how the systemic immune macroenvironment is modulated by neurosurgical tumor resection is particularly important in the context that it has been indicated tumor immune macroenvironment is remarkably plastic.^13^ In the present study, we found multidimensional modulation of systemic immunity by neurosurgical tumor resection involving both cellular and humoral immunity, which is consistent with previous findings about the plasticity of the systemic immune macroenvironment. And our study has further indicated that Days 1–3 after surgery belong to the immunologically vulnerable period of time since both cellular and humoral immunity are significantly inhibited at this time point. In addition, patients a lower preoperative CD4+/CD8+ lymphocyte ratio subsequent develop postoperative infection complications, representing a susceptible population for opportunistic infection. These data indicate that the state of the patients, at the time following surgical resection, may be a critical determinant of whether the operation cures the patients or the complications develop. In general, a better understanding of the systemic consequences of surgery‐related immune remodeling is critical to improving the design and conduct of translational research and improving the outcomes of patients with brain tumors.

Recent studies from peripheral cancer in both experimental and clinical literature have provided a deeper understanding of the impact of surgical tumor resection on systemic immunity. Both detrimental and beneficial effects of surgery–tumor–host interactions have been supported by a growing body of evidence and gradually accepted by researchers and surgeons in this area.^9^, ^11^, ^22^ Overall, the detrimental effects of surgery include risk of tumor cells shedding into circulation,^23^ angiogenesis stimulation,^24^ new‐occurred and accelerated metastatic outgrowth following resection of the primary tumor.^12^, ^25^, ^26^ In contrast, the beneficial effects of surgery mainly involve reduced tumor burden, potential remodeling of the disturbed immune state in host harboring cancer, and reduced risk of metastasis. Our present study explored the effects of surgical resection of brain tumors in terms of dynamically systemic cellular and humoral immunity and also found the surgical dual characters. Differential modulation in systemic cellular and humoral immunity after surgery can be seen in our study. Either CD3+ lymphocytes, CD4+ lymphocytes, or CD4 + /CD8 + lymphocyte ratio synchronously presented bidirectional changes after surgery showing an initial decrease and then subsequent increase even more than the preoperative levels. Different weights of tissue damage responding to surgery and reduced tumor burden, and comprehensive interactions across the time following surgery, may explain the temporal reshaping of systemic immunity. Moreover, other lymphocytes such as NK lymphocytes and B lymphocytes show unidirectional changes thorough the entire observation period after surgery. In addition, in contrast to the increase in B lymphocytes frequency following surgery, immunoglobulins (IgA, IgG, and IgM) levels secreted from B lymphocytes decreased postoperatively. The suppression of immunoglobulins is thought to occur as a result of a primary inhibition of B lymphocyte function, or due to impairment of T cell function, which in turn inhibits the clonal expansion of B‐lymphocytes. Our findings suggest that neurosurgical tumor resection dynamically reshapes the composition of the immune macroenvironment after surgery. When targeting immune behavior therapeutically, it is particularly important to take the systemic immune state of host into account, appropriately combine conventional therapies such as surgery with immune modulation, and consider the immunologically vulnerable period following surgery or vulnerable patient population.

It should be noted that there are some limitations in the present study. First, the present study focused on depicting the perioperative composition of the immune macroenvironment in patients with brain tumors. The underlying mechanisms dedicated to the differentiate remodeling of systemic immunity by neurosurgical tumor resection did not be explored in the present study. Further research have been initiated in our center to answer this question. Second, the data of 49 patients were included for the present study, involving gliomas, brain metastases, meningiomas, and skull base tumors. Limited sample size makes the generalizations of our findings limited. In the future, well‐designed studies with greater sample size across the tumor type, tumor stage, tumor grade, and therapeutical combination are necessary. Finally, the present study is a retrospective study. Considering the accessibility, convenience and cost–benefit analysis among the assessing methods, lymphocyte subsets, serum immunoglobulins, CRP, and complements levels are routinely measured in patients with brain tumors during perioperative period in our institute. That is why these biomarkers are retracted from the medical records of patients to represent systemic cellular and humoral immunity. Other more targeted, complex, and sensitive biomarkers can be used in future prospective studies.

## 5 CONCLUSION

In conclusion, the present study provides evidence that neurosurgical tumor resection is associated with multidimensional changes in systemic immunity across the time after surgery and immune composition. Future studies are needed to determine the mechanisms behind systemic immune remodeling, whether the immune dysfunction can be prevented and restored, and whether the infectious morbidity after surgery can be avoided.

## AUTHOR CONTRIBUTIONS

Jia‐Wei Wang, Liujiazi Shao, and Jing‐Hai Wan conceived and designed the study. Jia‐Wei Wang, Hong‐Liang Wang, Ke Hu, Liujiazi Shao, and Qi Liu performed the data collection and statistical analyses. Jia‐Wei Wang drafted the initial manuscript. Liujiazi Shao and Jing‐Hai Wan made critical comments and revision for the initial manuscript. All authors reviewed and approved the final manuscript.

## CONFLICT OF INTEREST

The authors declare no conflict of interest.

## ETHICS STATEMENT

This research was approved by the Ethics Committee of the Cancer Hospital, Chinese Academy of Medical Sciences (No.22/052‐3253). All methods in this study were carried out in accordance with relevant guidelines and regulations.

## Data Availability

The datasets generated and/or analyzed during the current study are available from the corresponding author on reasonable request.
